# Identification of HLA-A*11:01 and A*02:01-Restricted EBV Peptides Using HLA Peptidomics

**DOI:** 10.3390/v16050669

**Published:** 2024-04-25

**Authors:** Yufei Wang, Wanlin Zhang, Ruona Shi, Yanran Luo, Zhenhuan Feng, Yanhong Chen, Qiuting Zhang, Yan Zhou, Jingtong Liang, Xiaoping Ye, Qisheng Feng, Xiaofei Zhang, Miao Xu

**Affiliations:** 1 State Key Laboratory of Oncology in South China, Guangdong Key Laboratory of Nasopharyngeal Carcinoma Diagnosis and Therapy, Guangdong Provincial Clinical Research Center for Cancer, Sun Yat-sen University Cancer Center, Guangzhou 510060, China; wangyf@sysucc.org.cn (Y.W.); zhangwl2@sysucc.org.cn (W.Z.); luoyr@sysucc.org.cn (Y.L.); chenyh2@sysucc.org.cn (Y.C.); zhangqt@sysucc.org.cn (Q.Z.); zhouyan1@sysucc.org.cn (Y.Z.); liangjt2@sysucc.org.cn (J.L.); yexp@sysucc.org.cn (X.Y.); fengqsh@sysucc.org.cn (Q.F.); 2 Guangdong Provincial Key Laboratory of Stem Cell and Regenerative Medicine, Guangdong-Hong Kong Joint Laboratory for Stem Cell and Regenerative Medicine, Center for Cell Lineage and Development, Guangzhou Institutes of Biomedicine and Health, Chinese Academy of Sciences, Guangzhou 510530, China; shi_ruona@gibh.ac.cn (R.S.); feng_zhenhuan@gibh.ac.cn (Z.F.); 3 Joint School of Life Sciences, Guangzhou Institutes of Biomedicine and Health, Chinese Academy of Sciences, Guangzhou Medical University, Guangzhou 511436, China

**Keywords:** Epstein-Barr Virus, epitopes, HLA peptidomics, nasopharyngeal carcinoma

## Abstract

Epstein-Barr Virus (EBV) is closely linked to nasopharyngeal carcinoma (NPC), notably prevalent in southern China. Although type II latency of EBV plays a crucial role in the development of NPC, some lytic genes and intermittent reactivation are also critical for viral propagation and tumor progression. Since T cell-mediated immunity is effective in targeted killing of EBV-positive cells, it is important to identify EBV-derived peptides presented by highly prevalent human leukocyte antigen class I (HLA-I) molecules throughout the EBV life cycle. Here, we constructed an EBV-positive NPC cell model to evaluate the presentation of EBV lytic phase peptides on streptavidin-tagged specific HLA-I molecules. Utilizing a mass spectrometry (LC-MS/MS)-based immunopeptidomic approach, we characterized eleven novel EBV peptides as well as two previously identified peptides. Furthermore, we determined these peptides were immunogenic and could stimulate PBMCs from EBV VCA/NA-IgA positive donors in an NPC endemic southern Chinese population. Overall, this work demonstrates that highly prevalent HLA-I-specific EBV peptides can be captured and functionally presented to elicit immune responses in an in vitro model, which provides insight into the epitopes presented during EBV lytic cycle and reactivation. It expands the range of viral targets for potential NPC early diagnosis and treatment.

## 1. Introduction

The Epstein-Barr Virus (EBV), a type of gamma herpesvirus that infects over 95% of adults worldwide [1], is strongly associated with nasopharyngeal carcinoma (NPC) since EBV is present in almost all NPC cases worldwide [2]. The EBV in NPC shows a type II Latency expressing several viral oncoproteins, including Epstein-Barr nuclear antigen 1(EBNA1), latent membrane protein 1 (LMP-1), latent membrane protein 2 (LMP-2A), and non-coding RNAs (Epstein Barr virus-encoded small RNAs (EBERs) and microRNA-BARTs) [3]. Despite the dominant contribution of EBV’s latent infection to tumorigenesis [4], the lytic phase of EBV still plays a crucial role in NPC [5]. Recent transcriptomic analysis has detected that several lytic genes are expressed in NPC biopsies, revealing a non-stringent EBV type II latent gene expression [6]. The lytic cycle is a process initiated by the expression of immediate-early (IE) genes, which express transcription factors for initiating viral replication. This is followed by the expression of early (E) genes which facilitate viral DNA replication, and late (L) genes responsible for the assembly and release of viral particles [7]. Genes in the early lytic phase are essential for viral propagation and have been implicated in promoting tumor progression [8], immune evasion, and possibly in the initial transformation of nasopharyngeal epithelial cells [9]. The examination of EBV gene expression in clinical NPC samples revealed the expression of type III latency and several lytic genes [10], including BZLF1 [11], BRLF1, BMLF [12] and BALF4 [13], etc.

EBV in NPC can sometimes exhibit abortive lytic activity with the expression of IE genes and some early genes such as BMRF1 and BALF2, and cells entering these phases may demonstrate an enhanced capacity of immune evasion and angiogenesis [14]. Additionally, the expression of these lytic cycle genes contributes to tumor growth and invasion by altering the immune environment, leading to immunosuppression and increased invasiveness in the advanced stages of NPC [9]. Furthermore, the elevated levels of antibodies targeting lytic antigens have been correlated with tumor progression in NPC patients. Recent research also showed that IgA and IgG antibodies targeting all EBV life cycle stages were associated with NPC [15], which may shed light on the unique pathogenic and therapeutic processes for NPC.

EBV-specific T cells have emerged as a promising target for controlling virus-infected cells and influencing the onset and progression of NPC [16]. Pioneering research has demonstrated that EBV-specific T cells can precisely target and eradicate EBV-positive cancer cells in vitro and in vivo [17,18,19]. These specialized T cells are adept at recognizing and reacting to viral antigens presented by major histocompatibility complex (MHC) class I molecules on the surface of infected cells [20], highlighting a critical mechanism for immune-mediated tumor clearance. However, the path to optimizing such a vaccine or immunotherapy is fraught with challenges, predominantly due to the limited knowledge of the precise antigens presented on the surface of NPC cells [21]. This gap significantly hampers the identification of ideal targets for therapeutic intervention [22]. One of the critical factors is the variability in HLA distribution, particularly in South China. This region exhibits a high global incidence of NPC and boasts a population with significant HLA diversity [23]. Notably, there is a high proportion of the HLA-A*11:01 allele distribution in this area. However, the restriction of EBV epitopes to this subtype is lacking, which underscores the need for targeted research to understand how this subtype influences antigen presentation and T cell recognition in NPC, as well as which EBV antigens can be presented on the surface of NPC cells [24]. HLA-A*02:01 is one of the most prevalent HLA alleles, commonly found in all ethnic populations and expressed at considerably high frequencies in Asian and African populations [25]. Although the presentation of EBV-derived peptides by HLA-A*02:01 has been widely studied [26], there is still a lack of epitopes, specifically during the lytic phase.

Epitope-prediction algorithms can be used to quickly predict the binding of peptides to a diverse array of HLA alleles. One limitation of this approach is that the algorithms mainly predict the binding of peptides [27], with their accuracy relying on the available data for specific HLA restrictions and the amino acids in the anchor residues [28]. Immunopeptidome is a reaching technology with the advance of high-resolution mass spectrometry (MS) [29,30,31]. Mass spectrometry has emerged as a powerful tool with soaring sensitivity that can assess the processing and presentation of precisely defined minimal epitopes at the cell surface [32]. Recently, several neoepitopes have been identified by MS [33], showing the feasibility of this approach. The widely utilized pan-HLA antibody method enables the direct identification of all presented HLA class I peptides. However, determining the peptides’ HLA restriction relies heavily on computational prediction, which represents a notable limitation [31]. Previous reports have indicated that tumor antigens, presented by streptavidin-tagged HLA, can be isolated explicitly without confusion by non-cancer cells [34]. Thus, to eliminate the need for in silico HLA-deconvolution, we used streptavidin-tagged HLA to isolate HLA-tagged peptide-MHC complexes directly. This approach allowed us to accurately pinpoint the specific peptides presented by HLAs of interest, thereby enhancing the confidence in directly determining the peptides displayed on the MHC-peptide complexes. By employing the strep-tagged HLA method and targeting specific HLA subtypes, we isolated predominant East Asian HLAs: HLA-A*11:01 and HLA-A*02:01 allele-restricted EBV epitopes from various NPC cell lines and validated their immunogenicity in EBV positive healthy donors, thereby expanding the EBV epitope database. We have identified several highly immunogenic peptides from EBV primarily in the early lytic phase, which could offer new insights and potential targets for early diagnosis or T-cell-based immunotherapy.

## 2. Materials and Methods

### 2.1. Cell Lines

EBV-positive human nasopharyngeal cancer cell lines C666 were cultured in RPMI-1640 medium (Thermo Fisher Scientific, Waltham, MA, USA) supplemented with 10% fetal bovine serum (FBS) and penicillin-streptomycin solution (100 mg/mL). Engineered HK1-Akata and CNE2-M81 cell lines harboring the recombinant EBV strain Akata-EBV-GFP and M81-EBV-GFP genome were propagated in RPMI 1640 containing 10% FBS, penicillin, and streptomycin and maintained under G418 (700 μg/mL) or Hygromycin B (700 μg/mL) selection separately. 293T was cultured in DMEM (Thermo Fisher Scientific, Waltham, MA, USA) supplemented with 10% fetal bovine serum (FBS) and antibiotics (penicillin-streptomycin solution, 100 mg/mL).

### 2.2. Plasmids

pCDH-CMV-Puro lentivirus vector was used to encode each streptavidin-tagged HLA-I molecule. The sequences were obtained from hla.alleles.org: HLA-A*11:01 (HLA: HLA00043) and HLA-A*02:01 (HLA: HLA00005). A fusion construct was generated to facilitate the effective binding between the exogenous HLA heavy chain and beta-2 microglobulin (β2m), whereby β2m was co-expressed with HLA. The construct comprises a signal peptide from the HLA heavy chain (residues 1–24), followed by the fusion structure. For the purpose of fusion expression, the coding sequence of β2m(297 bp in length) was engineered to be linked at the N-terminus of the HLA-A*11:01 molecule. A Twin-Strep tag II (SA-WSHPQFEK-(GGGS)_2_-GGSA-WSHPQFEK) was positioned at the C-terminus of the fusion protein to allow for downstream processes. psPAX2 and pMD2.G were used for lentivirus packaging.

### 2.3. Generation of HLA-A*11:01/HLA-A*02:01-Transfected NPC Cell Lines

293T cells were plated using T75 flasks at a seeding density of 7.5–9 × 10^6^ cells for lentivirus production. The transfection mix was comprised of pCDH-CMV-Puro-HLA-A*11:01 or HLA-A*02:01 with packaging plasmids (psPAX2 and pMD2.G) to a total ratio of 5:3:2. The mixture was added to the cells and the medium was changed after 6 h. Harvested viral supernatants at 24 and 48 h were further concentrated using a 10 kDa ultracentrifuge tube (Millipore, Billerica, MA, USA) at 4 °C for virus concentration. NPC cells were pre-plated in a six-well plate and mixed with lentivirus at a multiplicity of infection (MOI) 1. Polybrene was added to the cell-virus mixture at a final 6 μg/mL concentration. Puromycin selection started on day three after transduction at 1 μg/mL concentration. 

### 2.4. Immunopeptidome Sample Preparation and Collection

EBV-positive cell lines carrying the recombinant EBV (CNE2-M81, HK1-Akata) were served as 48H group and induced by 20 ng/mL 12-O-tetradecanoylphobol 13-acetate (TPA;Yeasen, Shanghai, China) and 2.5 mM sodium butyrate (NaB; Sigma Aldrich, Saint Louis, MO, USA) for 12 h followed by media exchange. After 48 h in culture, the cells were washed twice with PBS, then collected and centrifuged to get cell pellets. C666 cells with endogenous EBV expression were collected in their innate state. Non-inductive cell lines were categorized as 0H (without TPA and NaB treatment). Cells were washed twice with PBS, collected, and centrifuged to obtain cell pellets.

### 2.5. MHC-Peptide Immunoprecipitation

To isolate Strep-tagged HLA-I peptides, frozen cell pellets expressing strep-tagged HLA molecules were placed on ice to allow complete thawing. Cells were lysed with 5 × cell pellet volumes of lysis buffer containing 1 × freshly added protease inhibitor (4693132001, Roche, Indianapolis, IN, USA) on a rotator at 4 °C for 1 h. Post-lysis, the samples were centrifuged at 20,000× *g* for 20 min at 4 °C, with the supernatant collected for BCA quantification (23225, Thermo Fisher Scientific, Waltham, MA, USA). From each sample, 3 mg of protein lysate was taken and combined with 30 μL of Strep-Tactin beads (2-1204-001, IBA, Goettingen, Germany) for Strep-tagged specific IP. Following a binding period of 1.5 h at 4 °C, the samples underwent three washes with cell lysis buffer and two washes with 50 mM Tris (pH 8.0). The supernatant was then fully aspirated, and the beads were eluted by shaking at 25 °C at 1500 rpm for 2 min with 50 μL of 10% acetic acid. The eluate was collected, and the elution process was repeated once. The combined eluates were then passed through a 10 kDa cut-off column (MRCPRT010, Millipore, Billerica, MA, USA). The flow-through was vacuum-concentrated to dryness, reconstituted in 100 μL of 0.1% formic acid, and the pH was adjusted to below 2 using 10% TFA. Desalting was then performed using a homemade stage-Tip, after which the peptides were concentrated and prepared for subsequent analysis.

### 2.6. Western Blot

Protein concentration was quantified with BCA, and samples were loaded onto 10% SDS-PAGE gels and electrophoresed at 120 V until the loading dye reached the bottom of the gel. Gels were then transferred onto the PVDF membrane at 300 mA for 2.5 h. Blots were blocked with 5% non-fat milk in PBST for 1 h at room temperature and then incubated with the indicated primary antibody overnight at 4 °C. HRP-conjugated secondary anti-mouse antibody (A90-116P, Bethyl, Montgomery, TX, USA) were used for 2 h at room temperature. 5 times PBST washing was applied in between every step. The image was visualized using the ChemiDoc^TM^ system with BeyoECL Plus developing buffer (P0018, Beyotime, Shanghai, China). Images were processed with Biorad Image Lab Software (Version 6.1, Hercules, CA, USA) and Image J (Version 1.54, Bethesda, MD, USA). Primary antibodies used in this study include anti-pan-HLA (W/632, Biolegend, San Diego, CA, USA) or Strep-Tactin HRP (2-1502-001, IBA, Goettingen, Germany).

### 2.7. LC-MS/MS and Data Analysis

Peptides were analyzed using reversed-phase ultra-high performance liquid chromatography (EASY-nLC1200) coupled with Orbitrap Fusion Lumos mass spectrometer (Thermo Fisher Scientific, Waltham, MA, USA) equipped with a nano-electrospray ion source. The instrument settings were optimized as follows: the spray voltage was set to 2200 V, and the capillary temperature was maintained at 325 °C. Full-scan mass spectrometry (MS) analyses were performed over a mass-to-charge ratio (*m*/*z*) range of 300–1800. The resolution for MS1 scans was set at 120,000 (at *m*/*z* 200), with a maximum injection time of 120 ms to ensure adequate ion sampling. The Automatic Gain Control (AGC) target was adjusted to 500,000 ions to optimize the signal intensity and data accuracy. The RF lens strength was set to 40% to enhance ion transmission efficiency. Peptide precursor ions were isolated using a narrow isolation window of 1.2 *m*/*z* to minimize interference from adjacent ions. The instrument was configured to include charge states ranging from 2 to 6 for MS/MS analysis, enhancing the detection of multiply charged peptides commonly observed in protein digestion mixtures. Dynamic exclusion was applied with a duration of 30 s to prevent the repetitive selection of previously analyzed precursor ions, thereby improving the diversity of peptide identifications during the LC-MS/MS run. Resulting spectra were processed and analyzed using fragpipe. Database search used the built-in HLA peptide search (Nonspecific-HLA) workflow with recommended settings for HLA peptides. The peptide length was restricted to 7-25aa. MSFragger search assumes cysteines were not alkylated. Mass tolerance for processing was set to 5 ppm for precursor ions and 0.5 Da for fragment ions. The enzyme digestion was set to unspecific. Cysteinylation (C +119) is specified as a variable modification. PSM is filtered to 1% FDR. MSBooster for MS/MS spectral and RT-based rescoring, in conjunction with Philosopher, all integrated into the Fragpipe workflow. After getting peptide lists, the length filtration was first done combined with the HLA binding prediction were done using NetMHCpan-4.1 (percentile rank score ≤ 2%). The logo plots were generated by seq2logo. LC-MS fragment spectra was generated using pLabel. The measured mass of the precursor peptide ion ([M + 2H]^2+^), charge state (z), and the retention time (RT) at which the peptide ion was selected for fragmentation are stated within each spectral panel. C-terminal fragment ions are indicated as y, N-terminal fragments are designated b. 

### 2.8. Peptide Synthesis

All EBV Peptides were successfully synthesized at GenScript (purity > 95%). Synthetic peptides were dissolved in dimethyl sulfoxide (DMSO) at 20 mg/mL and stored at −80 °C. 

### 2.9. Human Peripheral Blood Mononuclear Cell (PBMC) Isolation and HLA Typing

Whole blood was collected from 16 EBV serum VCA- and EBNA1-IgA positive high-risk healthy donors from Wuzhou, China. Ficoll-Paque was used for PBMCs and granulocyte isolation using density gradient centrifugation. The red blood cells in the cell sediments were lysed using ACK lysis buffer. Purified cells were frozen separately until use. DNA was isolated from 1 × 10^6^ granulocytes using the DNeasy Blood & Tissue Kit (Qiagen, Germantown, MD, USA), following the manufacturer’s instructions. The HLA subtype of donors was determined by the sequencing center (iGeneTech, Beijing, China). 

### 2.10. Peptide-Specific T Cell Line Generation

PBMCs derived from EBV carriers were resuspended at a concentration of 1 × 10^6^ cells/mL in R10 medium. Depending on the determined or known HLA types and the number of available cells, HLA-matching peptides and controls were added to individual wells. To prepare the stimulators, a third of the aliquot was washed in FCS-free RPMI-1640 media and incubated with single peptides (10 μg/mL) for 2 h, washed once, resuspended, and mixed with the remaining two-thirds of the cells, which were allocated as responders. Stimulators and responders were mixed and incubated for 3 days at 37 °C with 5% CO_2_. On Day 3, the medium was partially replaced with one containing 25 U/mL recombinant human IL-2 (200-02, PeproTech, Waltham, MA, USA), and 20 ng/mL each of IL-7 (R&D, Minneapolis, MN, USA) and IL-15 (200-15, PeproTech), with half-media changes every two days. On Day 5, cells were stimulated again with autologous PBMCs preloaded with peptide. IL-2 was added to achieve a final concentration of 50 U/mL. After 14 days, peptide-specific CD8^+^ T cells were harvested for cell count and viability assessment.

### 2.11. IFN-γ ELISPOT Assays

IFN-γ ELISpots were performed using Human IFN gamma ELISpot KIT (BD ELISPOT, Franklin Lakes, NJ, USA), following the manufacturer’s instructions. Peptides were used at a final concentration of 1 μg/mL per peptide as negative controls were used DMSO. phytohemagglutinin (PHA) was used as a positive control. For ex vivo assays, we used a total of 5 × 10^4^ cells EBV-specific T cells per well. Plates were precoated overnight with an anti-IFN-γ monoclonal antibody (mAb). After incubation for 16 to 20 h at 37 °C in 5% CO_2_, the secondary biotinylated anti-IFN-γ antibody was added to the plates. Following incubation with streptavidin-horseradish peroxidase (HRP), the reactions were developed with the 3-amino-9-ethylcarbazole (AEC) substrate (AEC substrate set, BD ELISPOT). Plates were analyzed using the CTL ImmunoSpot Image Analyzer (ImmunoSpot, Cleveland, OH, USA) and ImmunoSpot software (Version 7.0.15.1). The results are shown as spots per well and were considered positive if they were equal or greater than 5 spots and at least 2 times above the means of the unstimulated control wells.

### 2.12. Flow Cytometry

For surface expression makers, NPC cells stably expressing tagged-HLA were analyzed using pan-HLA antibody (W6/32, Biolegend, San Diego, CA, USA), anti-HLA-A*11:01-Biotin antibody (clone 8.L.170, Mirus, Madison, WI, USA) followed by Streptavidin-APC second antibody (BD), or anti-HLA-A*02:01-PE (clone BB7.2, Biolegend, San Diego, CA, USA). For intracellular strep-tag staining, cells were fixed and permeabilized with 1× fixation buffer/permeabilization wash buffer (BioLegend) and stained with the following Streptavidin-APC antibody (BD). Cells were acquired on a CytoFLEX S instrument (Beckman Coulter, Krefeld, Germany), and the data were analyzed using FlowJo software 10.8.1.

## 3. Results

### 3.1. Subsection

**Figure 1:**
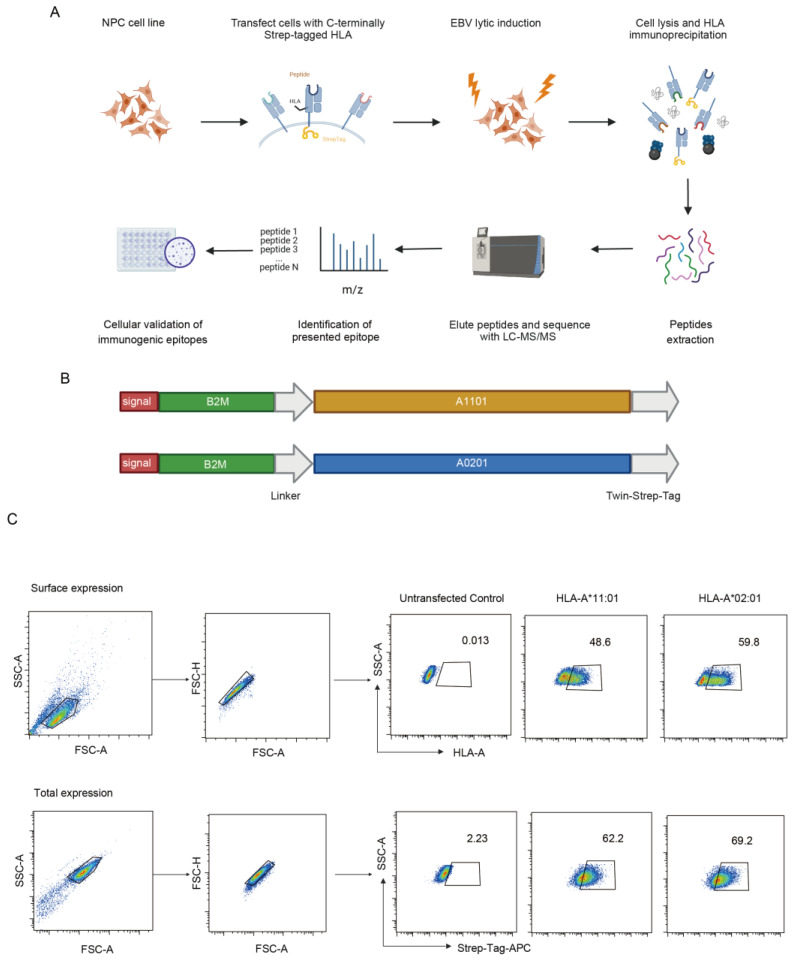
Pipeline of EBV immunopeptidome and validation of strep-tagged HLA overexpression. Overview of the strep-tagged HLA overexpression in NPC cell lines to discover EBV epitopes using LC-MS/MS and in vitro immunogenicity test. (**A**) NPC cell lines were engineered to express streptavidin-tagged HLA-A*11:01 or HLA-A*02:01. After induction, EBV-positive cell lines were collected for the isolation of immunopeptidome to identify EBV peptides. (**B**) Vector construction for strep-Tagged HLA expression. Twin-streptavidin tags (gray) were added at the C-terminus of each HLA molecule, which was coupled with beta-2 microglobulin (green) through a fusion expression by a linker (gray). Signal peptides were derived from the HLA heavy chain (red). (**C**) HLA surface and total expression were validated by flow cytometry. Representative pseudocolor plots from the HK1-akata cell line are shown. Untransfected cell line is the control.

To get EBV epitopes from NPC cell line, we used the immunopeptidomic workflow as depicted (Figure 1A). This process began with the construction of the cell line, providing a solid platform for subsequent analyses. After establishing the cell line, we induced the lytic phase of EBV within these cells. Immunoprecipitation was then performed to isolate peptide-MHC molecules from the harvested cell samples. The separated peptides were subjected to mass spectrometry for identification to get a clear snapshot of the viral peptide repertoire. Finally, we evaluated the immunogenicity of these peptides.

#### 3.1.1. Construction of Cell Line with Tagged HLA

The repertoire of peptides presented by HLAs on the cancer cellular surface is crucial for immune surveillance especially for CD8^+^ T cell targeting, which relies on HLA-restricted recognition and killing. To get this important immunogenic signal to generate and sustain an effective T cell response for EBV, initially, the EBV-positive NPC cell lines CNE2, HK1, and C666 were selected. C666 is unique as it harbors endogenous EBV genomes during long-term culture and passage [35], whereas CNE2 and HK1 cell lines were engineered to stably harbor the Akata or M81 EBV-BACmid (bacterial artificial chromosome, BAC). C666 and M81 EBV genomes are derived from NPC cells and are the NPC-high-risk EBV strains. They carry high-risk variants strongly linked with NPC progression and are more lytic with enhanced tropism for epithelial cells [36,37]. In contrast, the Akata EBV genome is derived from Burkitt lymphoma and is not considered as a risk strain for NPC. Despite the large population in South China where HLA-A*11:01 is predominant, only a handful of HLA-A*11:01-restricted CD8^+^ T cell epitopes have been identified for EBV [38]. Thus, we embarked on an epitope discovery study to define more EBV epitopes potentially relevant for future therapeutic applications. Additionally, the HLA-A*02:01 subtype was selected due to its considerable population coverage worldwide and extensive documentation in scientific research. 

We engineered HLA-A*11:01 and HLA-A*02:01 molecules by adding a twin-streptavidin-tag at the C-terminus of each HLA molecule coupled with beta-2 microglobulin (β2M) fusion expression to enhance stability (Figure 1B). These modified HLAs were transfected separately into different EBV-positive NPC cell lines. By exploiting the twin-strep-tag’s strong affinity and high specificity, we aimed to isolate a more significant number of relevant targets. After a week of selection with puromycin, stable HLA-expressing cell lines were established, and the surface expression of these alleles was verified using flow cytometry. We measured the surface expression by staining with HLA-A*11:01 and HLA-A*02:01 antibodies and the anti-strep-tag antibody, respectively. Following permeabilization of the transfected alleles, we assessed both surface and total expression. The results showed great congruency, demonstrating that more than 50% of the cells were positive for the markers (Figure 1C; Supplementary Figure S1).

In conclusion, we have successfully constructed NPC cell lines that efficiently express tagged HLA-A*11:01 and HLA-A*02:01, which can be detected by streptavidin antibody.

#### 3.1.2. Isolation and Identification of EBV Peptides Presented on NPC Cell Lines

EBV exhibits latent expression patterns in NPC. However, lytic gene expression also holds great significance for tumor oncogenesis and progression [13]. Previous reports have shown that in certain conditions when the lytic cycle is triggered in NPC, sequential expression of immediate-early, early genes and limited late lytic proteins can be expressed [8]. To investigate EBV life cycle dynamics within NPC and capture the EBV peptides presented, we collected cells in which EBV was in its latent phase, as well as the early lytic phase, which are both essential periods for understanding the virus-host interactions. The EBV induction protocol for EBV-positive epithelial cell lines was adjusted by modulating TPA and NaB stimulation. Lytic-induced cells were harvested 48 h post-stimulation to primarily focus on early lytic activities, given their greater significance in tumor expression profile. Non-induced cell lines where EBV is maintained in the latent phase were harvested at the same time. To avoid ambiguity, samples that have not undergone induced lytic activity (no treatment) were consistently labeled as 0H in this paper. All the cell lines collected for further immunopeptidomics analysis are listed below (Table 1).

Having confirmed the expression levels of HLA and successfully constructed the EBV latency and lytic replication model, we harvested approximately 1 × 10^8^ cells per sample and performed immunopeptidomics experiments for all cell lines in duplicates. HLA-bound peptides for each engineered cell line were first isolated by HLA immunoprecipitation using strep beads. Elutes were then validated by Western blot, confirming the successful isolation of HLA-I α-chains and their fusion with β2M, yielding a combined molecular weight of approximately 60 kDa, without major contaminations by other cellular proteins. Purified peptide samples were analyzed by high-resolution LC-MS/MS using a state-of-the-art mass spectrometer. Subsequent database searches were conducted with MSFragger against the relevant EBV viral antigens (Akata, M81, and C666-1, respectively) and human Uniprot proteome in the same analysis. This was followed by the MSBooster and Philosopher, all integrated into the Fragpipe workflow. Following spectral identification, high-confidence hits were filtered based on a false discovery rate (FDR) of 1%.

**Figure 2:**
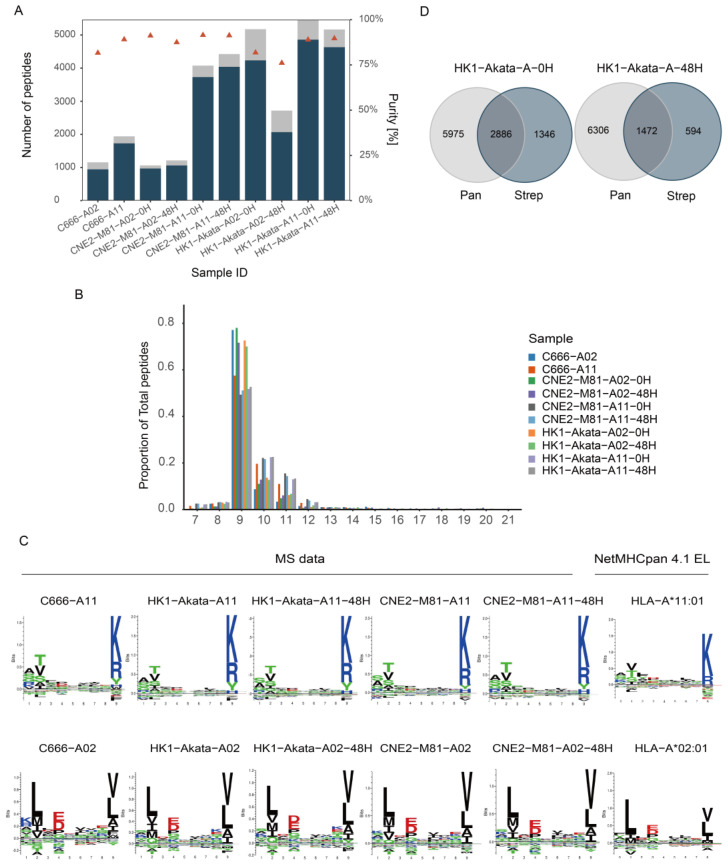
Characteristics of identified peptides. (**A**) Yields of isolated peptides for different cell lines achieved by LC-MS/MS. Peptide yields varied between 1106 and 5830 (mean = 3422) per sample. The purity is defined as the proportion of binders among all identified peptides and indicated by triangles on the right axis. Total peptides were shown as a gray bar, and those binding to a specific allele as a blue bar. (**B**) Typical length distribution of all eluted peptides in proportion to total identifications from different samples. (**C**) 9-mer peptides clustered to reveal the main binding motifs fit the correlating HLA subtypes. (**D**) Overlap of peptide sequences between pan-HLA(W6/32) IP and strep IP.

LC-MS/MS-based analysis of the HLA class I ligandome identified a median of 3422 peptides per sample, with a range from 1106 to 5830 peptides. Although the same amount of cells were collected, the number of peptides identified varied between samples. The purity was assessed by estimating the affinity of the eluted peptides to the respective HLA molecules to predict binding affinities (using NetMHCpan4.1). Our eluted peptides showed at least 80% purity (a percentage of peptides have a solid predicted binding motif matching the HLA subtype and were predicted as strong or weak binders) (Figure 2A). The lengths of identified peptides followed the characteristic length distribution of HLA-I peptides, and the majority of peptides exhibited a length of 9 amino acids, with a considerable proportion of 8mer, 10mer, and 11mer peptides (Figure 2B). Submission of the 9mer peptides from all different samples for binding motifs analysis showed the motifs are compatible with classic HLA-peptides binding motif and the specific HLA-A*11:01 and A*02:01 anchor residue as shown in previous mono-allelic cell line research (Figure 2C).

Besides, this strep-tag-based for identifying peptide-MHC complexes method was proved to get allele-specific HLA presented peptide as widely used W6/32 method by comparing the co-currency of peptides from HLA-Akata-A02 with or without EBV lytic induction (Figure 2D). The concordance rates obtained by different immunoprecipitation methods were above 70%, showing a quite solid approach to getting allele-specific peptides.

#### 3.1.3. Detection of High Confident EBV Epitopes

As anticipated, we could not identify any EBV peptides in the non-lytic NPC cell line. EBV in these cells was in a latency phase, where antigens are rarely expressed or expressed at relatively low levels and seldom presented on the cell surface [39]. We detected several EBV peptides in lytic NPC cell lines. Among the EBV peptides identified, two peptides ATIGTAMYK (HLA-A*11:01 restricted) from BRLF1 [40] and TLDYKPLSV (HLA-A*02:01 restricted) from BMRF1 [41] were previously reported and listed in the IEDB, which further confirmed the credibility of our results. Overall, we identified eight peptides derived from the EBV M81 strain and five from the Akata strain, among these five were restricted to HLA-A*11:01 and eight to HLA-A*02:01.

These peptides are integral to various proteins in the virus’s life cycle, including one immediate-early lytic peptide, ten early lytic peptides, and two late peptides. Among the early lytic peptides, we have identified two derived from BMRF1—a protein crucial for viral DNA replication. Its presence in patient tissues and cell lines suggests a significant role in the pathogenesis of NPC [10]. Additionally, we have discovered a peptide from BALF2, involved in DNA packaging potentially associated with NPC progression through complex interactions within the tumor microenvironment [9]. Other early lytic peptides belong to proteins such as BLLF3, BFLF1, BORF2, BKRF3, BRRF1, and BSLF1, though the exact roles were less defined in NPC, are important for the virus’s maturation [42] and also play roles in immune evasion and modulation of the host’s defense mechanism [14]. For late lytic phase peptides associated with the BBRF1 protein, essential for virion assembly and maintaining the viral capsid structure, while the BPLF1 protein, an early lytic enzyme with deubiquitinating activity that aids the virus in manipulating the host cell environment to promote viral replication and survival [43].

**Figure 3:**
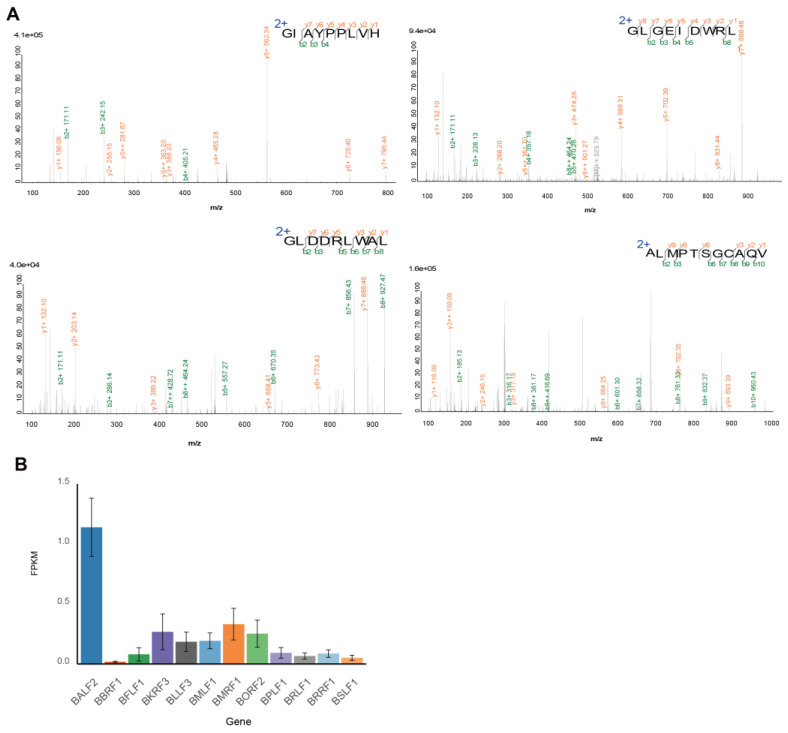
EBV peptides presented profile and gene expression profile. (**A**) LC-MS fragment spectra are shown as measured in the experiment. (**B**) RNA transcript levels for EBV genes, shown in different colors, were indicated in 276 NPC tumor samples. Data are represented as mean ± S.E.M.

We confirmed the spectra show a high confidence albeit in low intensity, due to the low expression level and presentation of EBV antigen (Figure 3A). Using RNA-seq data from NPC tumor samples, we also confirmed that all the peptides-derived genes we detected were expressed in the NPC tumor samples consistent with literature reports [44], which also showed a non-restricted type II latency expression, suggesting they may play a pathogenic role and have potential for future applications (Figure 3B).

In summary, we found thirteen peptides from different EBV antigens: one from the immediate early phase, ten from the early lytic phase, and two from the late lytic phase. The peptides and origin of cells are listed below (Table 2).

#### 3.1.4. Immunogenicity of Identified EBV Peptides

**Figure 4:**
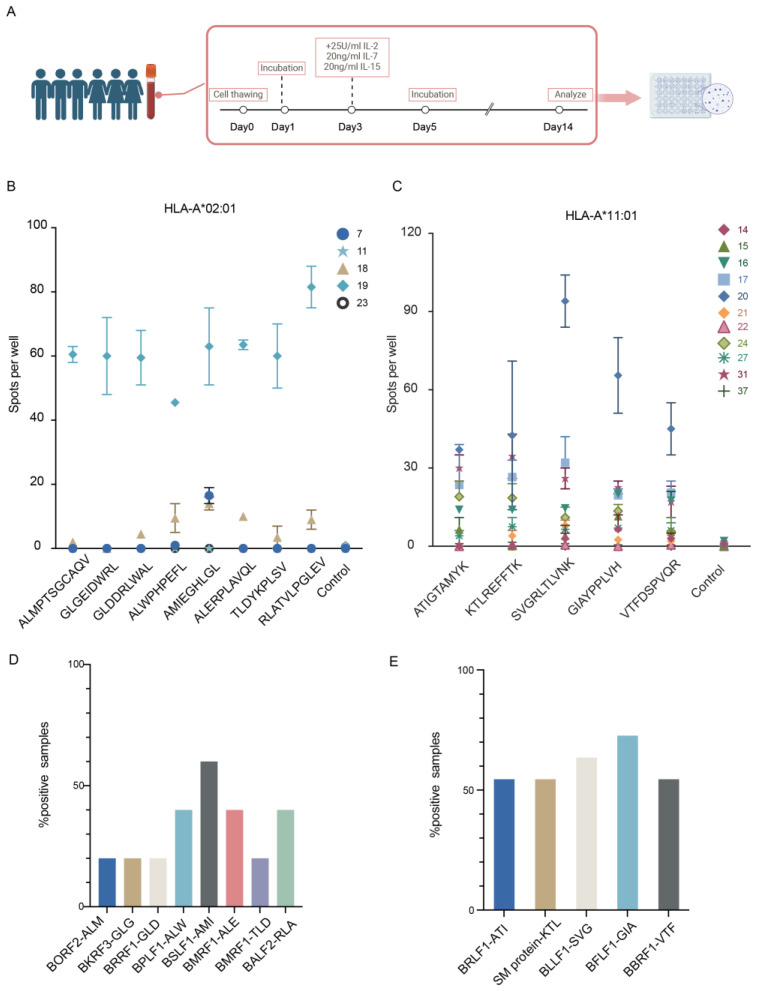
Validation of immunogenicity of identified peptides in EBV-positive healthy donors. (**A**) Experimental workflow. (**B**) Frequency of IFN-γ^+^ CD8^+^ T cells following stimulation with HLA-A*02:01 EBV peptides. (**C**) Frequency of IFN-γ^+^ CD8^+^ T cells following stimulation with HLA-A*11:01 EBV peptides. In panels B and C, the means ± SEMs are shown (*n* = 2 biological replicates) (**D**) Percentage of HLA-A*02:01 positive donors responding to the peptide. (**E**) Percentage of HLA-A*11:01 positive donors responding to the peptide.

To validate the identified EBV peptides possess the immunogenic potential capable of eliciting HLA-A*11:01 and HLA-A*02:01-restricted CD8^+^ T cell responses, we choose five healthy donors carrying HLA-A*02:01 allele and eleven donors harboring HLA-A*11:01 allele. PBMCs were harvested from these EBV seropositive healthy donors came from Guangxi Wuzhou and showed EBV viral capsid antigens-IgA (VCA-IgA) and nuclear antigen 1-IgA (EBNA1-IgA) positive. Since serum EBV EBNA-1 and VCA-IgA antibodies can be detected in 3–5 years before the diagnosis of NPC and have been widely used for NPC screening in mainland China and Taiwan, these donors were categorized as a high-risk population for the development of NPC [45,46,47]. Short-term CD8^+^ T cell lines were generated by stimulation with different peptides for 14 days, followed by re-stimulation with the cognate peptide to measure interferon-gamma (IFN-γ) production using IFN-γ ELISPOT assays (Figure 4A). Different donors had varying degrees of response. The previously published epitopes showed great immunogenicity, as we expected. The frequencies of IFN-γ^+^ CD8^+^ T cells were significantly higher than those in the respective DMSO negative and blank control. Three of the five donors carrying HLA-A*02:01 allele showed responses that were above the basal IFN-γ CD8^+^ T cell frequencies, especially for donor 19, probably showing a strong EBV reactivation in this donor. The RLA-BALF2 epitopes elicited the strongest response, while the late ALW-BPLF1 epitope had a relatively lower response. Donor 18 showed reactions to several peptides including BMRF1, where our newly discovered peptide elicited a response despite the reported one showing no reaction (Figure 4B). Besides, For the HLA-A*11:01 peptides, all of them could elicit IFN-γ response in peptide-specific T cells. Among the HLA-A*11:01 restricted donors, five out of eleven donors showed reactions to all peptides, especially donor 20, showing a particularly strong reaction to SVG-BLLF3 (Figure 4C). The HLA-A*11:01 donors had a higher overall frequency of IFN-γ positive donors reactive to corresponding peptide stimulation (Figure 4D,E). Our results suggest that in an NPC high-risk population, further testing for antibodies or cellular immunity against some lytic genes might allow for more precise subgrouping within the population and identification of more relevant screening markers.

In conclusion, we detected a T cell response to each peptide sequence in at least one donor, confirming that the mass spectrometry identified peptides were immunogenic in the context of natural infection and confirmed that the peptides detected using our method is indeed immunogenic epitopes.

## 4. Discussion

In our study, we used a comprehensive antigen discovery approach aimed at enhancing our understanding of the immune response against EBV in NPC progression. We leveraged the high-affinity benefits of strep-tag purification to reduce noise and background in the eluted peptides. The utilization of strep-tagged HLA overexpression in EBV-positive NPC cells allowed for a refined discovery of thirteen EBV peptides presented on highly extensive HLA-A*11:01 and HLA-A*02:01 subtypes. We evaluated the immunogenicity of these peptides in EBV-IgA-positive populations and showed their potential to elicit an immune response. In conclusion, our study contributes to the database of epitopes in EBV, which are important additional targets to be considered for future diagnostic and immunotherapeutic approaches to nasopharyngeal carcinoma.

The majority of EBV T cell epitopes identified to date have been discovered through immunogenicity screenings across various donors or by utilizing overlapping peptide pools to pinpoint specific epitopes of interest [48]. The Immune Epitope Database (IEDB) lists a total of 409 EBV epitopes, predominantly restricted to HLAs common in Western populations, yet there is a noticeable scarcity of epitopes associated with the dominant HLA subtypes in Southeast Asia. Particularly, HLA-A*11:01 is prevalent in this region, with an allele frequency of about 30%, yet there are only five EBV epitopes identified that are restricted to A*11:01. To augment the repository of A*11:01-restricted EBV epitopes, we employed an HLA overexpression technique to uncover additional EBV epitopes specific to A*11:01 and found one published epitope and four novel epitopes restricted to A*11:01 with immunogenicity. Moreover, though many epitopes remain undiscovered, the identification of several new HLA-A*02:01 epitopes has helped us bridge the current gap in identifying more EBV epitopes. Additionally, it is important to mention that since we are using an NPC cell line model, the endogenous HLA may affect the presentation by the transfected HLA. However, as we observed in our preliminary data, both endogenous and exogenous HLA can present a significant number of peptides as they appear to exhibit random loading onto these alleles. Furthermore, since immunopeptidome can detect peptides of high abundance, those present at high levels can be detected by both the pan-HLA antibody method and our strep-tagged method. Further studies are needed to either knock down the endogenous HLA or use cell lines carrying irrelevant HLAs to obtain more detailed information. 

The lytic phase of EBV in NPC has not been thoroughly studied, as it is commonly believed that EBV remains dormant in NPC. Yet, an increasing number of studies demonstrate the significant role of the lytic phase in NPC progression [8,9,14]. Unlike the complete lytic cycle, where the virus finishes its replication and releases new virions, the abortive lytic phase is marked by partial viral replication with the activation of selected lytic genes. This phase holds particular importance in the context of NPC, where cells with the expression of lytic proteins can reshape the tumor microenvironment, influence tumor cell behavior, and potentially adjust the host immune response. Among the population we tested, individuals with A*11:01 allele exhibited more responses, while only some with A*02:01 showed strong reactivity. The inter-individual variability raises questions about whether these enhanced immune responses provide stronger protection or as an indicative marker of potentially higher risks for NPC. Since novel biomarkers with high sensitivity for NPC screening arise, we focused on several lytic EBV epitopes, primarily in the early lytic phase around the EBV life cycle, which can provide new insight that could potentially serve as biomarkers or aid in prognostic assessments in NPC. This finding suggests the presence of EBV-specific T cells responsive to our identified lytic antigens, indicating their potential in early diagnosis and stratification of populations at high risk for NPC.

## 5. Conclusions

Together, our research employs an antigen discovery approach, encompassing HLA peptidome profiling, selection of candidate peptides, and evaluation of functional immunogenicity. By using our strep-tagged HLA overexpressing on EBV-positive nasopharyngeal carcinoma cells, we yielded thirteen EBV peptides presented on HLA-A*11:01, HLA-A*02:01 and assessed their immunogenicity in EBV-positive donors.

## Figures and Tables

**Table 1:** Sample collection.

Cell Line	HLA Class I Typing	EBV Strain	Strep-Tagged HLA Overexpression	Lytic Induction
CNE2	A*68:02:01;A*11:01:01	M81	A*11:01	non-lytic
				48H
			A*02:01	non-lytic
				48H
HK1	A*24:02:01;A*24:02:01	Akata	A*11:01	non-lytic
				48H
			A*02:01	non-lytic
				48H
C666	A*24:260;A*24:02:53	C666-1	A*11:01	non-lytic
			A*02:01	non-lytic

**Table 2:** Identified EBV epitopes.

Peptide	Length	Accession	HLA Restriction	EBV Cycle
**ATIGTAMYK**	9	EBV-M81-1|BRLF1	HLA-A*11:01	Immediate Early Lytic
KTLREFFTK	9	EBV-M81-1|SMprotein	HLA-A*11:01	Early Lytic
SVGRLTLVNK	10	EBV-M81-1|BLLF3	HLA-A*11:01	Early Lytic
GIAYPPLVH	9	EBV-M81-1|BFLF1	HLA-A*11:01	Early Lytic
ALMPTSGCAQV	11	EBV-M81-1|BORF2	HLA-A*02:01	Early Lytic
GLGEIDWRL	9	EBV-M81-1|BKRF3	HLA-A*02:01	Early Lytic
GLDDRLWAL	9	EBV-M81-1|BRRF1	HLA-A*02:01	Early Lytic
ALWPHPEFL	9	EBV-M81-1|BPLF1	HLA-A*02:01	Late Lytic
AMIEGHLGL	9	EBV-Akata|BSLF1	HLA-A*02:01	Early Lytic
ALERPLAVQL	10	EBV-Akata|BMRF1	HLA-A*02:01	Early Lytic
**TLDYKPLSV**	9	EBV-Akata|BMRF1	HLA-A*02:01	Early Lytic
RLATVLPGLEV	11	EBV-Akata|BALF2	HLA-A*02:01	Early Lytic
VTFDSPVQR	9	EBV-Akata|BBRF1	HLA-A*11:01	Late Lytic

## Data Availability

The data that support the findings of this study are available from the corresponding author upon reasonable request. The mass spectrometry proteomics data have been deposited to the ProteomeXchange Consortium via the PRIDE [49] partner repository with the dataset identifier PXD051538.

## Supplementary Materials

The following supporting information can be downloaded at: https://www.mdpi.com/article/10.3390/v16050669/s1, Figure S1: Surface expression of transfected HLAs. HLA-A*11:01 and HLA-A*02:01 surface expression was validated by flow cytometry for CNE2-M81 and C666 cell lines, with untranfected cell lines as the control.
