# Supplementary figures and images for: Identification of HLA-A*11:01 and A*02:01-Restricted EBV Peptides Using HLA Peptidomics
Source: Viruses. 2024 Apr 25;16(5):669. doi: 10.3390/v16050669 (PMC11125987; doi:10.3390/v16050669)

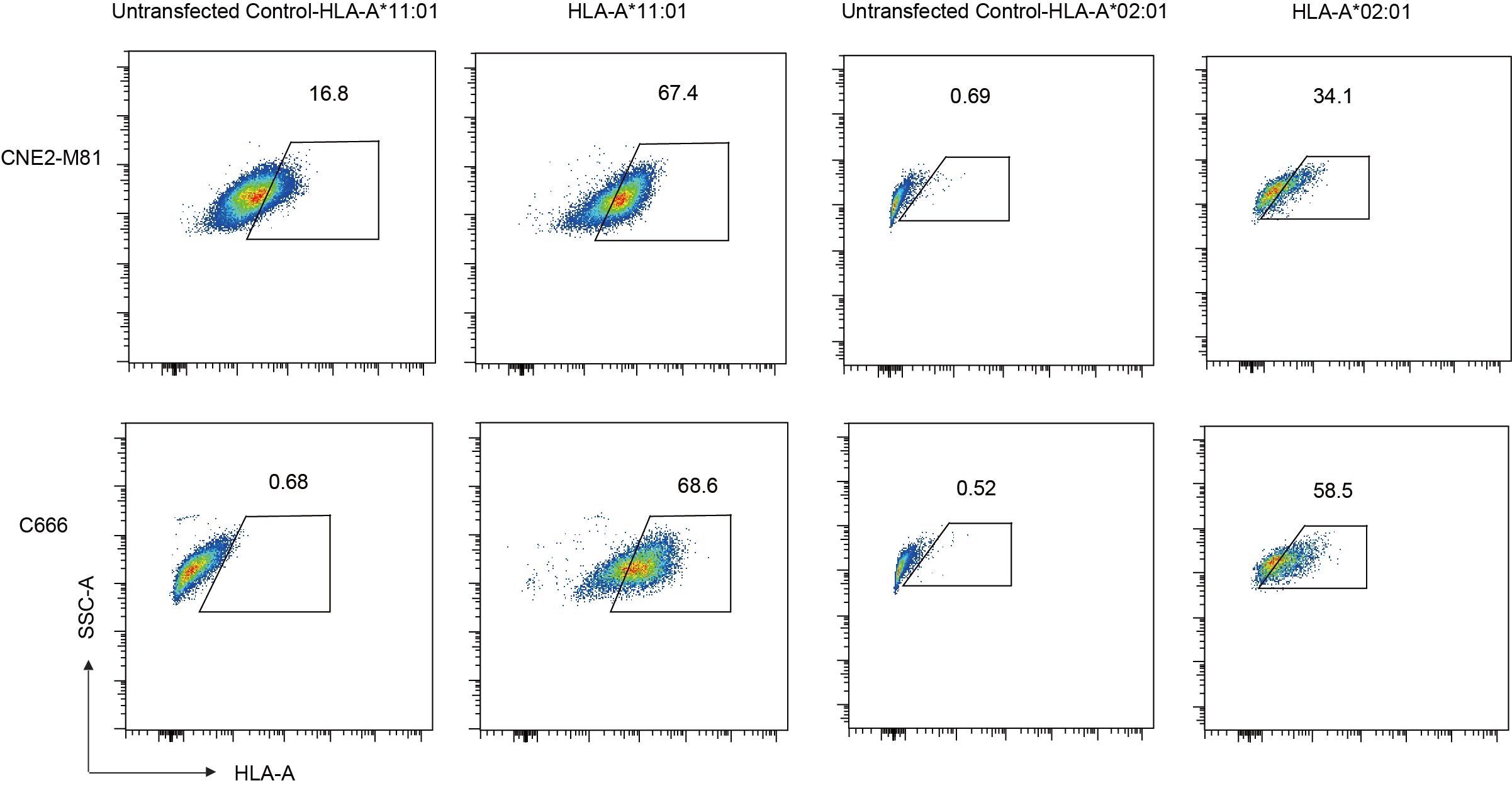

Supplement: Supplementary file 1 [file viruses-16-00669-s001.zip › viruses-2955058-supplementary.png]
